# Socioeconomic status and partaking in air pollution monitoring are associated with cookstove usage across three peri-urban communities in sub-Saharan Africa

**DOI:** 10.1038/s41598-025-11633-3

**Published:** 2025-07-16

**Authors:** Federico Lorenzetti, Emily Nix, Theresa Tawiah, Miranda Baame, Edna Sang, Emmanuel Betang, Daniel Wilson, Judith Mangeni, Ryan Chartier, Rachel Anderson de Cuevas, Reginald Quansah, Elisa Puzzolo, Bertrand Hugo Mbatchou Ngahane, Kwaku Poku Asante, Diana Menya, Daniel Pope, Matthew Shupler

**Affiliations:** 1 Department of Public Health, Policy and Systems, University of Liverpool, Liverpool, UK; 2 Kintampo Health Research Centre, Kintampo, Ghana; 3 Douala General Hospital, Douala, Cameroon; 4 School of Public Health, Moi University, Eldoret, Kenya; 5 Geocene, Berkeley, CA USA; 6 RTI International, Research Triangle Park, NC USA; 7 School of Public Health, University of Ghana, Accra, Ghana

**Keywords:** Stove use monitoring, Clean cooking, Liquefied petroleum gas, Sub-Saharan African, Time savings, Household air pollution monitoring, Energy and society, Energy access, Energy and behaviour, Energy economics, Energy efficiency, Energy justice, Energy management, Energy policy, Energy security

## Abstract

While transitioning from polluting cooking fuels (e.g. wood, charcoal) to cleaner fuels, like liquefied petroleum gas (LPG), can lead to time savings, the amount of time saved is uncertain due to minimal stove use monitoring (SUM) data. Approximately three months (mean:82 days (SD:41)) of SUM data from Geocene temperature sensors was collected from 186 households in Mbalmayo, Cameroon; Obuasi, Ghana and Eldoret, Kenya. Households exclusively using LPG (mean:1 h 22 min/day) cooked for two hours/day less than those stacking LPG and polluting fuels (3 h 19 min/day), and almost three hours/day less than those exclusively using polluting fuels (4 h 10 min/day). Financially insecure households exclusively using polluting fuels cooked for ~ 45 min longer (4 h 29 min) than financially secure households (3 h 45 min). During a 24-hour household air pollution (HAP) monitoring period, average cooking time was 38 min longer (3 h 48 min vs. 3 h 10 min) and households cooked nearly once more per day (3.63 events) than during the remaining SUM period (2.72 events). Longer cooking times among financially insecure polluting fuel users suggests that LPG access may disproportionately benefit poorer households via greater time savings. Households may cook for longer-than-normal when monitored for HAP.

## Introduction

Approximately three billion people, predominately in low- and middle-income countries (LMICs), depend on polluting fuels (e.g., wood, coal) for cooking and heating^1^. Transitioning to clean cooking fuels/energy, like liquefied petroleum gas (LPG), electricity and ethanol, can reduce exposure to household air pollution (HAP) and reduce the risk of several adverse health outcomes, including cardiopulmonary and respiratory diseases^2–4^. Large scale up of clean household energy can also greatly reduce unsustainable deforestation, as half of wood harvested worldwide is used as fuel^5^. Uptake of clean cooking fuels further positively impacts climate by reducing emissions of black carbon, a strong climate-pollutant emitted through biomass combustion^6^.

In addition to health and climate benefits, the time saved when primary cooks switch from polluting to clean cooking fuels is an important social benefit^7^. Time savings can especially benefit women and girls, who are typically tasked with cooking and gathering fuels due to gendered household roles^8,9^. The additional time gained by women can be reallocated toward other productive tasks, such as income-generating activities, which can foster their socioeconomic empowerment in a positive feedback loop^10^. For example, research conducted in an informal settlement in Nairobi, Kenya found that two-thirds of female household heads earned extra income from additional employment after switching from polluting fuels to LPG for cooking^11^.

Despite the potential social benefits associated with time savings, limited studies have quantified variation in cooking times between households using clean and polluting cooking fuels across different settings^12^. Studies that have assessed cooking time among different cooking fuel types have primarily relied on self-reported data from surveys, which is subject to recall and social desirability bias.

An objective measure of stove use, obtained from stove use monitors (SUMs) that measure stove temperature to quantitatively estimate cooking events and time, can overcome this limitation^13–15^. However, due to logistical and financial challenges, SUMs have typically been deployed over short time periods (e.g. 1–3 days). The cooking patterns captured by SUMs over a period a few days may not be representative of longer-term cooking trends, particularly if households may alter their cooking behaviours if they know they are being monitored^16^. Collecting longer-term SUM data (e.g. over several months) may help reduce potential biases in cooking time assessment as it will be more difficult for households to adjust their cooking patterns over an extended period.

Longer-term stove monitoring also increases the volume of data which can enable an assessment of other sociodemographic determinants of cooking time, such as income, education and household size. This information can help identify certain sub-groups that may cook for longer periods of time and may therefore benefit more from time savings when switching from polluting to clean cooking fuels. Such information may help policymakers, governments and community leaders prioritize certain households for clean household energy interventions and aid the development of appropriate clean energy programmes for maximizing the use of clean cooking fuels.

In this study, we report patterns of stove usage over a period of multiple months obtained from SUMs. The data was collected as part of the CLEAN-Air(Africa) programme, which was conducted across three peri-urban communities in sub-Saharan Africa^17^. We compare stove usage patterns between households exclusively using LPG, stacking LPG and polluting fuels and exclusively using polluting cooking fuels. We further analyse how stove use patterns vary between communities, by socioeconomic factors and when households are concurrently being monitored for household air pollution exposures.

## Methods

### Study setting

The study took place in Mbalmayo, Cameroon; Obuasi, Ghana; and Eldoret, Kenya. Mbalmayo is an agricultural town in the centre of Cameroon with around 60,000 residents and a tropical wet and dry climate with constant temperatures throughout the year. Obuasi is a mining community in the southern Ashanti region, with a population of approximately 175,000; that also experiences a tropical wet and dry climate with relatively stable temperatures. Eldoret is in the Rift Valley region of Kenya at an elevation above 2100 m with a population of around 475,000 and a subtropical highland climate with mild summers and cool winters.

### Study sample

In *Phase 1* of CLEAN-Air(Africa), a population-level survey on household fuel use characteristics was conducted among approximately 2,000 households in each of the three communities (see Shupler et al. 2021^18^ for further details). This survey provided a sampling frame for *Phase 2*, which compared cooking practices and self-rated health in households cooking primarily with LPG or exclusively with polluting fuels (around 400 households in each community)^19^. In *Phase 3*, a subset of households (~ 80 per community) who completed *Phase 2* were randomly selected to receive 24-hour household air pollution monitoring (more details are available in Shupler et al. 2024^20^) along with stove use monitoring. Stratified random sampling was again used to select roughly half of households cooking primarily with LPG and the other half cooking exclusively with polluting fuels. During the 24-hour household air pollution monitoring, field teams provided the same directions to participants in all three communities to maintain their daily cooking routines.

In Eldoret, Kenya, 25% (*n* = 14) of households also used their stoves for heating due to a milder climate in the mountainous region of Western Kenya. To ensure that all stove usage reported in the paper was due to cooking alone, we included only 42 households in Eldoret that reported using their stove solely for cooking. We conducted sensitivity analyses by calculating the total (cooking + heating) stove use time and compared to cooking time alone; these data are reported in the Supplement.

### Stove use monitoring

SUM was carried out for a period averaging approximately three months (length of SUM varied due to study logistics, such as fieldworker availability) among *Phase 3* households. During the SUM period, one day (randomly) coincided with a 24-hour household air pollution monitoring period^20^.

SUM was conducted with thermocouple temperature sensors provided by Geocene^21^. The thermocouples measured stove temperatures at 5-minute intervals and were placed on all cookstoves in the household. SUMs were placed approximately 15 cm from the centre of the flame to prevent the equipment from overheating, while still being able to detect temperature fluctuations. SUM data was downloaded by field team members at regular intervals, roughly every 1–2 months. Data was wirelessly uploaded to the Geocene cloud and monitored in real-time for quality control to check for potential errors and ensure proper placement of the SUM.

### Identifying cooking events

Cooking events were derived from the SUMs temperature data using the Geocene “FireFinder” proprietary algorithm. FireFinder examines the ‘slope’ of the temperature data to identity significant spikes and drops in stove temperature, which are labelled as the start and end of a stove use “event”, respectively. The algorithm is bounded by user-specified parameters describing the primary and minimum temperature thresholds for stove use and event duration. The temperature thresholds (primary and minimum) for defining cooking events were derived from indoor temperatures in study households measured by air pollution monitors (MicroPEMs)^20^ to determine the plausible range of ambient temperature fluctuations. Separate thresholds were developed for each community due to regional differences in ambient temperature (Supplementary Table 1). The minimum stove use event duration was set to five minutes and multiple stove use events registered to a SUM within 30 min of each other were grouped as a single stove use event. Negative temperatures and temperatures exceeding 800 °C were removed due to implausibility.

For households with SUM placed on multiple stoves concurrently, the temperature data was merged to avoid double counting; thus, a cooking event in the household occurred when at least one stove registered an event in the SUM data.

### Statistical analysis

We descriptively report means and standard deviations of daily cooking time and number of cooking events by community, cooking fuel type and sociodemographic characteristics. To assess statistical significance for differences in average daily cooking time and number of cooking events, we used two sample t-tests if the variable had two categories and analysis of variance (ANOVA) if there were more than two categories. Data analysis was carried out using Microsoft Excel and Python version 3.9, including the Pandas and Matplotlib libraries^22^.

### Ethical approval

All methods were performed in accordance with the relevant guidelines and regulations, and in compliance with the Declaration of Helsinki. This study received ethical approval from the University of Liverpool, United Kingdom, and local ethics committees in each study country: Central Regional Ethics Committee for Human Health Research (Cameroon), Institutional Research and Ethics Committee for Moi Teaching and Referral Hospital and Moi University (Kenya) and Kintampo Health Research Centre (Ghana). Informed written consent was obtained from all the participants prior to conducting the study.

## Results

A total of 186 households (80 Cameroon, 64 in Ghana and 42 in Kenya) were included in the analysis. Approximately 30% of study households exclusively used LPG, while 49% cooked only with polluting fuels. The remaining 22% of households used both LPG and polluting fuels.

As some households had more than one stove, a total of 342 stoves were monitored across the 186 homes (Table 1). SUM was conducted for an average of nearly three months (mean: 82 days; SD: 41 days). Approximately half (56%) of households had an LPG stove, 33% of households had a traditional non manufactured stove (three stone fire, chepkube (a locally built mud stove fuelled by wood), sawdust or wood chips) and 11% of households used a charcoal stove.

The mean age of the main cook was 34.6 years and 95% were female. An average of 5.82 members lived in the study households; the households contained an average of 2.9 rooms. Families tended to be larger in the Cameroonian setting, with a mean of 7 members, compared with a mean of approximately 5 household members in the Kenyan and Ghanian communities (Table 1). Participants in Mbalmayo were also substantially less likely to report being financially secure (11%) than primary cooks in Eldoret (38%) and Obuasi (30%).

Stove use locations varied substantially between communities. In Eldoret, most households (54%) used their stoves indoors in a separate room. In Mbalmayo, one-third of households used their stoves in a separate room that was partially open to the outdoors. In Obuasi, three-quarters of households placed their stoves outdoors on a veranda (Table 1).

**Table 1:** Summary statistics of study households by community.

Characteristic (*N* (%))	All	Eldoret, Kenya	Mbalmayo, Cameroon	Obuasi, Ghana
**Households monitored (N)**	186	42	80	64
**Fuel type**				
Exclusive LPG use	55 (30%)	16 (38%)	18 (22%)	21 (33%)
LPG and polluting fuels	40 (22%)	11 (26%)	24 (30%)	5 (8%)
Exclusive polluting fuel use	91 (49%)	15 (36%)	38 (48%)	38 (59%)
**Stoves monitored (N)**	342	67	145	130
**Stove types monitored**				
LPG burners	192 (56%)	43 (64%)	80 (55%)	69 (53%)
Three stone fire	69 (20%)	2 (3%)	54 (37%)	13 (10%)
Traditional non manufactured	45 (13%)	8 (12%)	2 (1%)	35 (27%)
Traditional manufactured	36 (11%)	14 (21%)	9 (6%)	13 (10%)
**Days of monitoring [mean (SD)]**	82 (41)	83 (63)	75 (23)	91 (39)
**Age of main cook [mean (SD)]**	34.6 (11.1)	31.33 (9.48)	37.54 (12.82)	33.05 (8.7)
**Female main cook**	176 (94.62%)	39 (92.86%)	75 (93.75%)	62 (96.88%)
**# of household members [mean (SD)]**	5.82 (2.94)	5.38(2.75)	7.1(3.02)	4.5(2.24)
**# of rooms [mean (SD)]**	2.94 (1.7)	3.24(1.85)	3.49(1.47)	2.05(1.53)
**Education of household head**				
No education	3 (2%)	0 (0%)	1 (1%)	2 (3%)
Primary	12 (6%)	2 (5%)	8 (10%)	2 (3%)
Secondary	88 (47%)	5 (12%)	59 (74%)	24 (38%)
University	26 (14%)	11 (26%)	12 (15%)	3 (5%)
Other/not specified	57 (31%)	24 (57%)	0 (0%)	33 (52%)
**Occupation of household head**				
Business owner	47(25%)	10 (24%)	17 (21%)	20 (31%)
Informal sector worker	7 (4%)	2 (5%)	1 (1%)	4 (6%)
Government/business employee	58 (31%)	21 (50%)	31 (39%)	6 (9%)
Craftsperson	15 (8%)	0 (0%)	5 (6%)	10 (16%)
Farmer	23 (12%)	9 (21%)	2 (2%)	12 (19%)
Unemployed/retired/family care	25 (13%)	0 (0%)	19 (24%)	6 (9%)
Other/not specified	11 (9%)	0 (0%)	5 (6%)	6 (9%)
**Cooking location**				
Indoors -no separate room	10 (5%)	4 (10%)	6 (8%)	0 (0%)
Indoors -separate room	50 (27%)	25 (60%)	14 (18%)	11 (17%)
Outside -separate room	41 (22%)	10 (24%)	28 (35%)	3 (5%)
Outside -on porch/open air	69 (37%)	3 (7%)	17 (21%)	49 (77%)
Other/not specified	16 (9%)	0 (0%)	15 (19%)	1 (2%)
**Financial security (sufficient income to meet weekly expenses)**				
Enough	44 (24%)	16 (38%)	9 (11%)	19 (30%)
Not quite enough	87 (47%)	21 (50%)	40 (50%)	26 (41%)
Definitely not enough	54 (29%)	5 (12%)	31 (39%)	18 (28%)

### Stove use patterns by fuel type

Overall, households used their stoves approximately three times per day on average (mean: 2.72 events, SD: 1.38). However, households stacking LPG and polluting fuels used their stove roughly 1.5 more times per day (mean: 3.62 events/day) than those exclusively using LPG (2.52 events/day) or exclusively using polluting fuels (2.45 events/day). At a community level, households cooking exclusively with LPG used their stoves less times per day in Eldoret (*p* = 0.057) and Mbalmayo (*p* = 0.002), but not in Obuasi (*p* = 0.200) (Table 2).

**Fig. 1:**
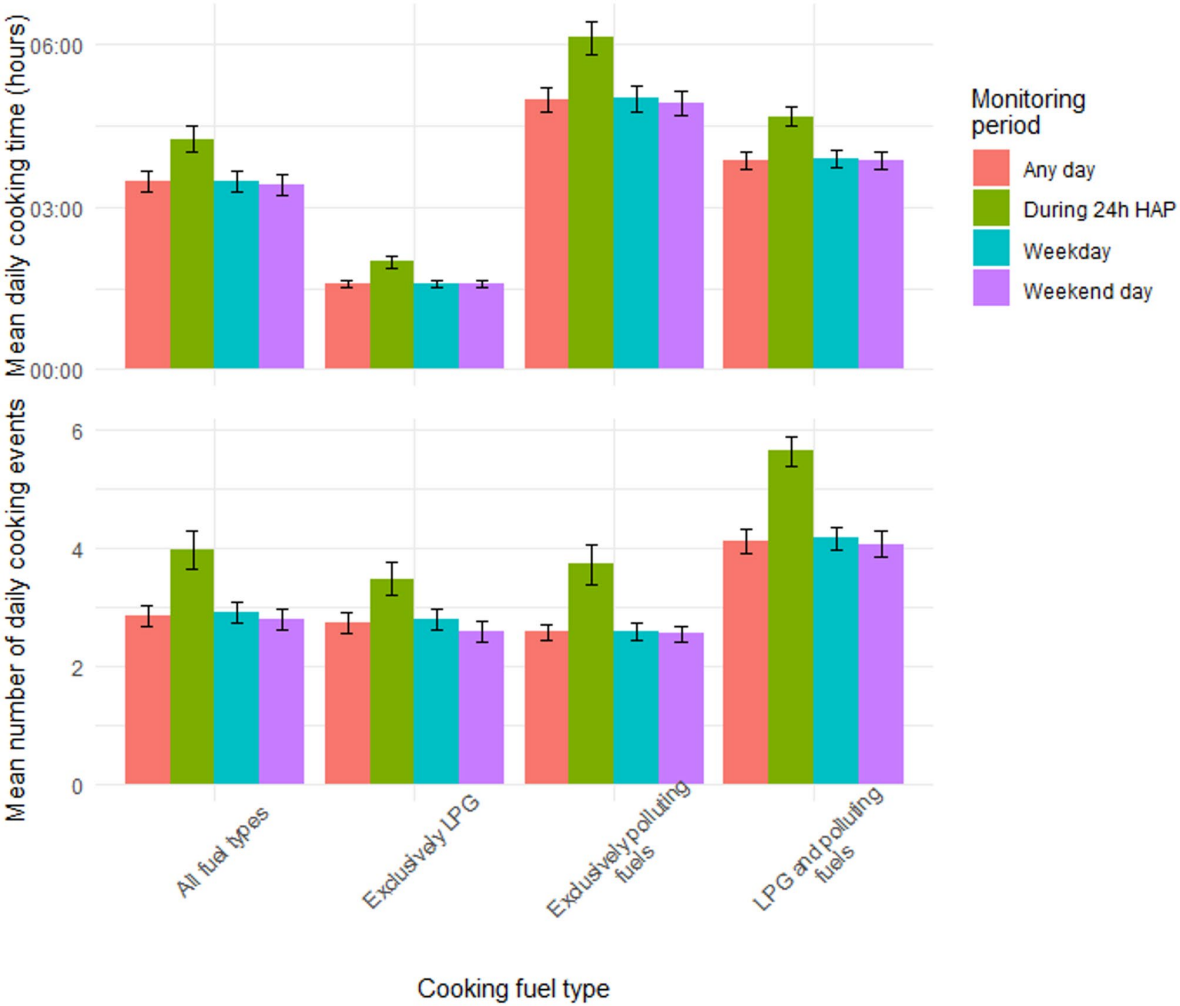
Stove use patterns across all communities by fuel type.

There was minimal variability in the mean number of stove use events on weekdays and weekends (Fig. 1). However, participants used their stove nearly one more time per day, on average, during the 24-hour household air pollution monitoring period (3.63 events/day; SD: 2.24) compared with the overall average daily use during the SUM period (2.73 events/day; S D: 1.38).

Households using only LPG had the shortest daily mean cooking time (1 h 22 min/day) (Fig. 1). Households stacking LPG and polluting fuels used their stoves for approximately two hours longer (mean: 3 h 19 min/day) than exclusive LPG users. Households using only polluting fuels used their stoves for an average of three hours per day longer (mean: 4 h 10 min/day) than those only cooking with LPG. In each community, households cooking exclusively with LPG cooked for significantly less time than those cooking exclusively with polluting fuels on weekdays and weekends (*p* < 0.001) (Table 2). During the 24-hour household air pollution monitoring period, only households in Eldoret and Mbalmayo that exclusively cooked with LPG cooked significantly less than households only cooking with polluting fuels (*p* < 0.001). In Obuasi, households cooking with polluting fuels decreased their average cooking time by approximately 50 min during the household air pollution monitoring period (from 3 h 15 min to 2 h 24 min), and did not cooking significantly more (*p* = 0.213) than households exclusively cooking with LPG (1 h 21 min) (Table 2).

Average daily cooking time did not substantially differ between weekdays and weekends (Fig. 1).

Across all communities, the mean cooking time during the 24-hour household air pollution monitoring period was 38 min longer (3 h 48 min/day) than the overall mean daily cooking time (3 h 10 min/day) when air pollution monitoring was not taking place (Fig. 1). However, the relative increase in mean daily cooking time during the household air pollution monitoring period varied by fuel type (Fig. 1). The mean daily stove use time among households exclusively using LPG was 27 min longer (1 h 49 min/day vs. 1 h 22 min/day) during the 24-hour household air pollution monitoring period compared with all other days during the SUM period. In comparison, the mean daily stove use time during the household air pollution monitoring period was ~ 40–50 min longer than the overall mean daily stove use time among households stacking LPG with polluting fuels (4 h 0 min/day vs. 3 h 19 min/day) and exclusively using polluting fuels (4 h 58 min/day vs. 4 h 10 min/day).

HAP = household air pollution.

### Community-level stove use patterns

The mean number of daily cooking events and cooking time also varied by community. Among households exclusively cooking with LPG, cooks in Eldoret used their stove an average of over three times per day (3.23 events/day), while those in Mbalmayo (2.81 events/day) and Obuasi (2.29 events/day) used their stove less than three times per day (Table 2). In Eldoret, those exclusively using polluting fuels used their stove more than three times per day (3.16 events/day), compared with less than three times per day in Obuasi (2.23 events/day) and Mbalmayo (2.40 events/day). In Eldoret, the average daily cooking time was over one hour longer (4 h 20 min), than daily cooking time in Mbalmayo (3 h 02 min) and almost two hours longer than the mean cooking time in Obuasi (2 h 34 min) (Table 2).

**Table 2:** Mean daily number of cooking events and cooking time by community, time frame and cooking fuel type.

Metric	Community	Time frame	All households	Exclusively LPG	LPG and polluting fuels	Exclusively polluting fuels	*p*-value^a^
**Number of daily cooking events (Mean (SD))**	**Eldoret**,** Kenya (***N*** = 42)**	**Any day**	3.23 (1.91)	2.54 (1.69)	4.31 (2.34)	3.16 (1.49)	0.057
		**Weekday**	3.38 (2.01)	2.71 (1.95)	4.46 (2.48)	3.3 (1.40)	0.081
		**Weekend day**	3.06 (1.85)	2.36 (1.57)	3.98 (2.15)	3.14 (1.66)	0.077
		**24 h HAP** ^**b**^	5.09 (1.90)	5.12 (2.20)	5.91 (1.99)	4.25 (1.23)	0.123
	**Mbalmayo**,** Cameroon (***N*** = 80)**	**Any day**	2.81 (1.10)	2.88 (1.16)	3.39 (1.10)	2.40 (0.91)	0.002*
		**Weekday**	2.83 (1.10)	2.95 (1.14)	3.42 (1.07)	2.41 (0.93)	0.001*
		**Weekend day**	2.77 (1.17)	2.71 (1.26)	3.35 (1.25)	2.43 (0.95)	0.009*
		**24 h HAP** ^**b**^	4.03 (1.99)	3.65 (2.24)	4.97 (1.95)	3.52 (1.69)	0.026*
	**Obuasi**,** Ghana (***N*** = 64)**	**Any day**	2.29 (1.17)	2.20 (1.23)	3.19 (1.41)	2.23 (1.08)	0.200
		**Weekday**	2.31 (1.20)	2.28 (1.29)	3.28 (1.39)	2.20 (1.10)	0.170
		**Weekend day**	2.21 (1.14)	1.99 (1.14)	2.97 (1.47)	2.23 (1.07)	0.217
		**24 h HAP** ^*^	2.17 (1.93)	2.15 (1.59)	3.00 (2.16)	2.05 (2.16)	0.662
**Daily cooking time (Mean (SD))**	**Eldoret**,** Kenya (***N*>** = 42)**	**Any day**	04:20 (04:19)	01:11 (00:55)	04:26 (03:22)	07:37 (04:46)	< 0.001*
		**Weekday**	04:21 (04:19)	01:11 (00:55)	04:25 (03:24)	07:40 (04:42)	< 0.001*
		**Weekend day**	04:22 (04:23)	01:13 (00:57)	04:27 (03:25)	07:53 (04:52)	< 0.001*
		**24 h HAP** ^**b**^	06:06 (04:21)	02:26 (01:11)	05:22 (03:06)	09:50 (04:16)	< 0.001*
	**Mbalmayo**,** Cameroon (***N*** = 80)**	**Any day**	03:02 (01:36)	01:34 (00:49)	02:59 (01:18)	03:46 (01:35)	< 0.001*
		**Weekday**	03:03 (01:37)	01:34 (00:52)	02:60 (01:18)	03:46 (01:36)	< 0.001*
		**Weekend day**	03:02 (01:39)	01:32 (00:45)	02:59 (01:21)	03:47 (01:39)	< 0.001*
		**24 h HAP** ^**b**^	04:04 (02:54)	02:06 (02:13)	03:39 (02:26)	05:20 (02:57)	0.001*
	**Obuasi**,** Ghana (***N*** = 64)**	**Any day**	02:34 (02:02)	01:22 (00:59)	02:26 (01:03)	03:14 (02:16)	0.002*
		**Weekday**	02:35 (02:07)	01:25 (01:06)	02:30 (01:10)	03:15 (02:21)	0.005*
		**Weekend day**	02:27 (01:53)	01:16 (00:48)	02:16 (00:46)	03:09 (02:06)	< 0.001*
		**24 h HAP** ^**b**^	01:58 (01:60)	01:21 (01:20)	02:12 (00:44)	02:24 (02:25)	0.213

a. P-value calculated from t-test if variable had two categories and analysis of variance (ANOVA) if more than two categories.

b. A 24-hour period during which households received concurrent stove use and household air pollution (HAP) monitoring.

* Significant at the 95% confidence level.

### Stove use patterns by community and fuel type

There was a minimal difference in mean daily time using an LPG stove across the three communities (range: 1 h 11 min – 1 h 34 min). However, among households exclusively using polluting fuels, cooks in Eldoret used their stoves for approximately twice as long (7 h 37 min) as those in Mbalmayo (3 h 46 min) and Obuasi (3 h 14 min) (Table 2). Thus, the difference in mean daily stove time between exclusive LPG and polluting fuel users varied substantially by community, ranging from around two hours (3 h 14 min vs. 1 h 22 min) in Obuasi to over six hours (7 h 37 min vs. 1 h 11 min) in Eldoret (Table 2).

### Stove use time during household air pollution monitoring

Households used their stove nearly two more times per day, on average, during the 24-hour household air pollution monitoring period than during the rest of the SUM period in Eldoret (5.09 events/day versus 3.23 events/day, respectively) (Table 2). In Mbalmayo, households used their stove over one more time per day (4.03 events/day versus 2.81 events/day, respectively). Conversely, households in Obuasi did not use their stove more often during the household air pollution monitoring period (2.17 events/day) than during the rest of the SUM period (2.29 events/day).

Households in Eldoret increased their daily cooking time by an average of over 1.5 h (from 4 h 20 min to 6 h 06 min) during the 24-hr household air pollution monitoring period; households in Mbalmayo increased their mean daily cooking time by one hour (from 3 h 2 min to 4 h 4 min). In contrast, cooking time in Obuasi was 36 min lower during the 24-hour period of household air pollution monitoring compared with the rest of the SUM period (from 2 h 34 min to 1 h 58 min). This led to a greater discrepancy in community-level average daily cooking times during the household air pollution monitoring period, with participants in Eldoret (6 h 6 min) cooking thrice as long as those in Obuasi (1 h 58 min).

Among households using LPG, the mean daily stove use time during the 24-hour period of household air pollution monitoring in Eldoret and Mbalmayo was 1 h 15 min and 32 min longer, respectively, than the mean daily cooking time during the rest of the SUM period (Table 2). The discrepancy in stove use time during the household air pollution monitoring period in these two communities was even larger among households exclusively using polluting fuels; the mean daily stove use time during the 24-hour period of household air pollution monitoring in Eldoret and Mbalmayo was 2 and 1.5 h longer, respectively, than the mean daily stove use time during the rest of the SUM period (Table 2).

Conversely, in Obuasi, there was minimal change in mean daily stove use time among households exclusively using LPG during the 24-hour household air pollution monitoring period; the mean stove use time among households using only polluting fuels was roughly 50 min less during the household air pollution monitoring period than during the rest of the SUM period.

### Stove use patterns by hour of day

**Fig. 2:**
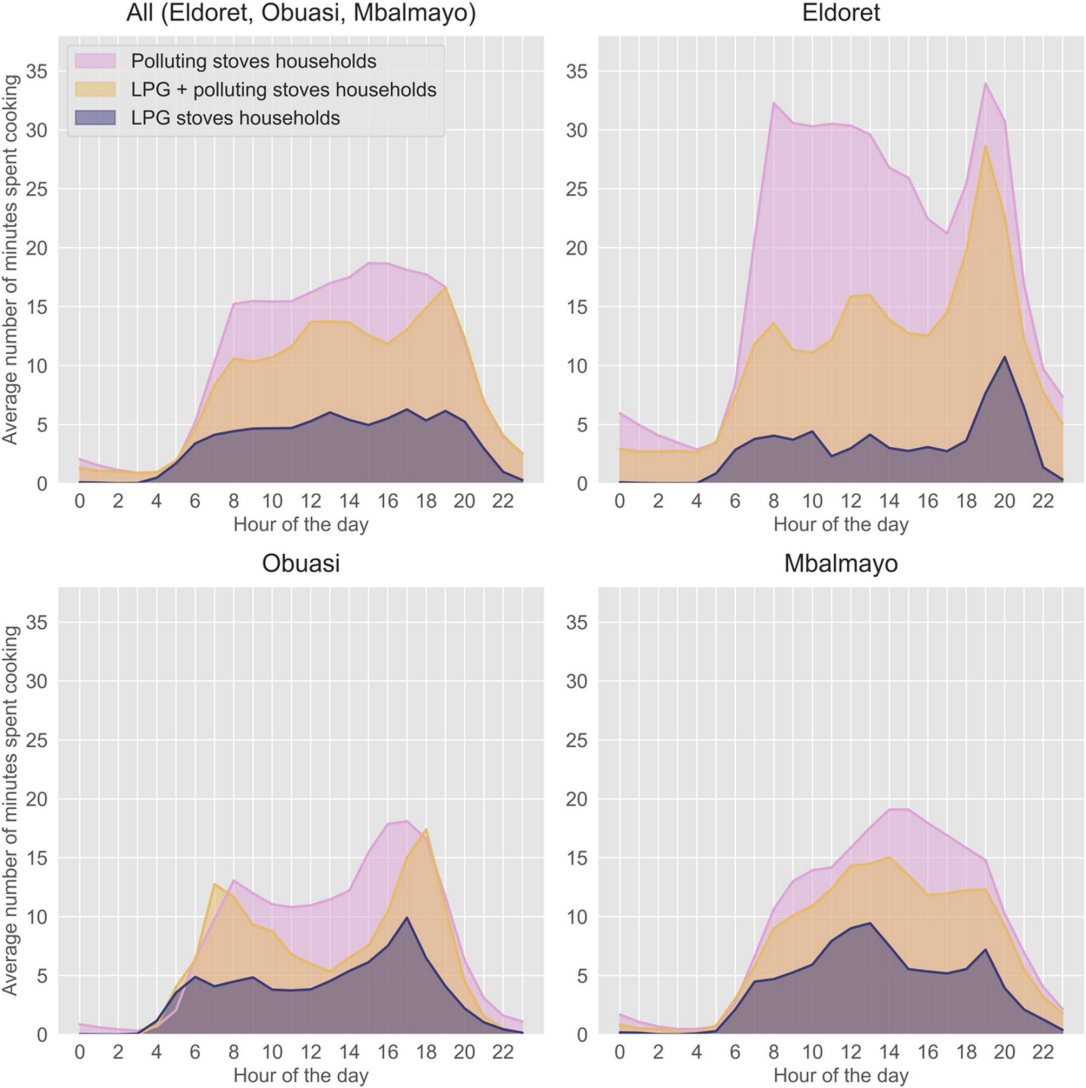
Daily variation in mean cooking time (minutes) by cooking fuel type and community.

In all communities, clear increases in stove use time occurred during breakfast (e.g. 7–8 am) and dinner hours (e.g. 6–8 pm) (Fig. 2). Cooking time also increased during lunchtime (e.g. 12 − 2 pm) in Mbalmayo and Eldoret but not in Obuasi (Fig. 2).

In Mbalmayo and Eldoret, households exclusively using polluting fuels used their stoves longer during each hour of the day than those stacking polluting fuels with LPG and only using LPG (Fig. 2). Contrastingly, in Obuasi, the mean stove time was longer during breakfast (e.g. 7–8 am) and dinner time (6–8 pm) among households using LPG and polluting fuels, compared to those only using polluting fuels (Fig. 2).

### Sociodemographic factors associated with mean daily stove use time

In addition to fuel type, sociodemographic factors were associated with mean daily cooking time. Among households exclusively using polluting fuels, a monotonically increasing relationship existed between increasing number of household members and mean daily stove use time; households with four family members or less used their stoves for around 3.5 h/day, on average, while households with 5–8 members used stoves for 4.5 h/day and those with 8 or more members for 5 h/day (Table 3). The rise in daily mean stove use time with increasing family size was largely driven by households in Eldoret. In Eldoret, the mean daily stove use time among households using exclusively polluting fuels with 3–4 members was 2 h 44 min, compared with 8 h 8 min among households with 5–8 members and 12 h 42 min among those with more than eight members (*p* = 0.062). In the other two communities, mean daily stove use time among families using only polluting fuels with more than eight members was approximately four hours (Table 3). The difference in mean daily stove use times between households with 3–4 members and 5–8 members in Obuasi (3 h 11 min and 3 h 07 min, respectively) and Mbalmayo (3 h 18 min and 3 h 38 min, respectively) was minimal (*p* = 0.820 and *p* = 0.506, respectively).

Among households exclusively using LPG, there was a lower difference in mean daily stove use time according to family size; mean daily stove use time among households with 1–2 members was 1 h 27 min, and mean stove use time among those with more than eight family members was 2 h 22 min (Table 4). Socioeconomic factors that impacted mean daily stove use time among exclusive LPG users were household ownership (rent vs. own) and financial security status. Similar to family size, this difference was mostly driven by participants in Eldoret; those owning homes used LPG nearly 30 min longer per day (1 h 32 min) as those renting their home (1 h 04 min) (Table 4).

The mean daily stove use time among households exclusively using LPG that reported not having enough money to meet their financial needs (56 min) was 30 min less than that among those that reported having enough money to meet their financial needs (1 h 23 min). Conversely, those who did not have enough money to meet their financial needs used polluting stoves for 45 min longer (4 h 29 min) than those that were financially secure (3 h 45 min) (Table 3). This trend existed across all three communities and was most evident in Eldoret, where the five families that reported ‘definitely not having enough money to meet their financial needs’ used their stoves for an average of over six hours longer (mean: 12 hr 33 min) than the 16 households that were financially secure (6 hr 37 min) (Table 3).

Across all communities, mean daily stove use time among households using polluting fuels in which an individual had received a secondary school education (3 h 46 min) was one hour and 30 min less than among those who received a primary school level education or had no education (5 h 21 min) (*p* = 0.079) (Table 3). The difference in mean daily cooking time among exclusive polluting fuel users that were secondary school educated (6 h 28 min) and received primary school education (8 h 33 min) was largest in Eldoret (difference of approximately 2 h).

In Mbalmayo (4 h 6 min) and Obuasi (3 h 18 min), households using polluting fuels outdoors used their stove longer per day than those that used stoves indoors (3 h 23 min and 1 h 59 min, respectively). The reverse was true in Eldoret, with households cooking with polluting fuels indoors (8 h 53 min) using their stove for over twice as long as those cooking outdoors (4 h 8 min) (Table 3).

**Table 3:** Mean daily cooking time by socioeconomic characteristics and community among households exclusively using polluting fuels.

**Characteristic**	All	p-value^a^	Eldoret, Kenya	p-value^a^	Obuasi, Ghana	p-value^a^	Mbalmayo, Cameroon	p-value^a^
Household size								
1–2	03:36 (02:57)	0.197	- (-)	0.062	02:57 (02:37)	0.820	- (-)	0.506
3–4	03:10 (01:41)		02:44 (01:39)		03:11 (01:60)		03:18 (00:56)	
5–8	04:28 (03:08)		08:08 (03:40)		03:07 (02:14)		03:38 (01:47)	
> 8	04:58 (03:44)		12:42 (08:51)		04:16 (03:19)		04:08 (01:35)	
Household ownership								
Own	05:09 (03:46)	0.004*	09:10 (04:33)	0.030*	03:51 (03:04)	0.124	03:43 (01:32)	0.757
Rent	03:11 (01:46)		03:21 (01:50)		02:29 (01:37)		03:53 (01:43)	
Enough money								
Enough	03:45 (03:14)	0.712	06:37 (05:31)	0.131	02:34 (01:35)	0.414	- (-)	0.859
Not quite enough	04:04 (02:30)		06:19 (03:29)		03:03 (01:55)		03:48 (01:30)	
Definitely not enough	04:29 (03:31)		12:33 (05:51)		03:53 (02:59)		03:43 (01:43)	
Cooking location								
Indoor	05:02 (03:55)	0.028*	08:53 (04:50)	0.088	01:59 (00:16)	0.436	03:23 (01:30)	0.164
Outdoor	03:35 (02:09)		04:08 (02:23)		03:18 (02:20)		04:06 (01:28)	
Education								
University	03:34 (01:46)	0.079	- (-)	0.458	- (-)	0.235	03:34 (01:46)	0.545
Secondary	03:46 (02:26)		06:28 (04:21)		02:50 (02:02)		03:54 (01:32)	
Primary/No Education	05:21 (04:19)		08:33 (05:27)		03:45 (02:34)		- (-)	

a. P-value calculated from t-test if variable had two categories and analysis of variance (ANOVA) if more than two categories.

* Significant at the 95% confidence level.

**Table 4:** Mean daily stove use time by socioeconomic characteristics and community among households exclusively using LPG.

Characteristic		All	*p*-value^a^	Eldoret, Kenya	*p*-value^a^	Obuasi, Ghana	*p*-value^a^	Mbalmayo, Cameroon	*p*-value^a^
**Household size**									
1–2		01:27 (00:48)	0.471	01:34 (00:32)	0.839	01:24 (00:56)	0.993	- (-)	0.2487
3–4		01:19 (00:49)		01:07 (00:42)		01:20 (00:50)		01:42 (01:00)	
5–8		01:20 (01:01)		01:10 (01:36)		01:23 (01:12)		01:21 (00:39)	
> 8		02:22 (01:09)		- (-)		- (-)		02:22 (01:09)	
**Household ownership**									
Own		01:28 (00:58)	0.576	01:32 (01:33)	0.395	01:22 (00:53)	0.970	01:31 (00:51)	0.796
Rent		01:20 (00:53)		01:04 (00:40)		01:23 (01:03)		01:37 (00:51)	
**Enough money**									
Enough		01:23 (00:58)	0.156	01:32 (01:16)	0.519	01:13 (00:48)	0.073	01:34 (01:07)	0.653
Not quite enough		01:35 (00:55)		01:07 (00:45)		02:06 (01:11)		01:42 (00:44)	
Definitely not enough		00:56 (00:39)		00:39 (00:45)		00:50 (00:37)		01:13 (00:44)	
**Cooking location**									
Indoor		01:22 (00:56)	0.811	01:16 (00:56)	0.506	01:05 (00:57)	0.318	01:47 (00:54)	0.352
Outdoor		01:25 (00:55)		00:51 (00:59)		01:34 (00:59)		01:23 (00:49)	
**Education**									
University		01:12 (00:39)	0.736	01:02 (00:38)	0.429	01:35 (00:12)	0.845	- (-)	0.947
Secondary		01:26 (01:03)		01:26 (01:18)		01:23 (01:03)		01:31 (00:60)	
Primary/No Education		01:24 (00:32)		- (-)		01:01 (01:03)		01:33 (00:13)	

a. P-value calculated from t-test if variable had two categories and analysis of variance (ANOVA) if more than two categories* Significant at the 95% confidence level.

## Discussion

This study uncovered varied drivers of stove use behaviours in peri-urban sub-Saharan Africa. We found that, despite using their stove approximately the same number of times each day, households exclusively using LPG cooked for over three hours less, on average, than those only using charcoal or wood (Fig. 1). This result is consistent with several other studies that reported the mean time spent cooking with LPG stoves is less than that of polluting cooking fuels^7^. There are numerous reasons why stove use can be less on LPG stoves compared with polluting fuels, including an instantaneous operation, an averted need to feed in biomass or tend to the fire and an improved ability to control the flame and therefore the speed of cooking^23^.

However, we note that the mean daily cooking time among households exclusively using LPG in our study (1 h 22 min) was less than half the median daily stove use time (3 h 52 min) registered to LPG stoves obtained from a multinational randomized controlled trial (also measured using SUMs) in rural communities of Peru, Guatemala, India and Rwanda^13^. In this trial, called the HAP Intervention Network (HAPIN), LPG stoves and fuel were provided at no cost, which removed the affordability barrier and may explain why households used the fuel for an average of over twice as long per day (in addition to potential dietary variations by geography). It is therefore possible that a desire to reduce LPG fuel expenditure is preventing households in our study from using LPG more frequently. Indeed, we find that financially insecure households used LPG for 30 min less per day than financially secure households (Table 4), suggesting that the cost of LPG may be affecting its frequency of use within the study communities.

We also find that financially insecure households used polluting fuels for an average of 45 min longer each day than financially secure households (Table 3). This implies that financially insecure households may experience greater time savings than financially secure households when switching from exclusively using polluting fuels to LPG. While intervention studies are needed to confirm the degree of time savings when switching from polluting to clean fuels, this study is one of the first to suggest that a transition from polluting fuels to LPG may help reduce socioeconomic inequities related to time poverty. A previous study conducted in urban Kenya found that informal sector workers were over 20% more likely to take on new income-generating activities after transitioning to LPG^11^, which also suggests that poorer households may experience greater socioeconomic benefits when transitioning to LPG.

We did not detect substantial variability in mean daily stove use times among exclusive LPG users across the three study communities at around approximately 1 h and 45 min per day (Table 2). This contrasts with the HAPIN trial, which found substantial variability in the median daily time spent cooking with LPG stoves (Guatemala: 4 h 59 min; Peru: 4 h 45 min; Rwanda: 3 h 51 min; India: 3 h 17 min)^13^. This may be explained by the affordability of LPG fuel being a potential limiting factor in the study communities and preventing households from using LPG as much as they would like. The sizable variability in LPG cooking times in the HAPIN trial, where fuel affordability did not impact use, may be due to differences in cooking requirements for the types of meals prepared and additional end uses such as water heating for bathing activities^24,25^.

Contrary to LPG usage, we did find considerable between-community variability in mean daily stove use times among exclusive polluting fuel users; households in Eldoret used their stove for roughly twice as long (mean: ~7.5 h/day) as those in Obuasi and Mbalmayo (mean: ~3.0–3.5 h/day) (Table 2). The longer stove time in Eldoret relative to Mbalmayo and Obuasi, particularly during the 24-hour household air pollution monitoring period (Table 2) may likely explain why Eldoret consistently had the highest household air pollution (PM_2.5_ and CO levels) out of the three communities^20^. The differences in mean daily stove use time between communities may be partly attributed to variations in the climate experienced or the types of foods cooked^24,25^. For example, some Kenyan households also used their stove for heating purposes due to the milder climate. Indeed, a previous study reported that space heating accounted for 40% of polluting fuel use^24^. While we excluded households from our analysis that reported also using their wood stoves for heating, some self-reporting bias may have existed and increased our reported cooking times in that community. Moreover, cooks in Obuasi were more likely to leave their home during the day than those in Eldoret and Mbalmayo^20^, and therefore may have been less likely to cook lunch at home (Fig. 1); this may partially explain why mean daily cooking times were lower in Obuasi compared with Eldoret and Mbalmayo.

Due to the community-level variability in stove use practices, the difference in mean daily stove use time between households exclusively using LPG and those only using polluting fuels varied four-fold, from around 1.5 h in Obuasi to nearly six hours in Eldoret. This suggests more stove monitoring is needed across communities to more accurately estimate potential time savings of a large-scale transition from polluting cooking fuels to clean cooking fuels.

### Multiple fuel use

This study finds that households using LPG and polluting fuels used their stoves more often (3.62 events/day) than households using only polluting fuels (2.45 events/day) or only LPG (2.52 events/day) (Fig. 1). This finding is supported by other literature that shows that the introduction of a new stove can increase the frequency of cooking since some households may prepare more complex meals that use more energy when they have access to multiple cooking devices^23,26^. However, despite households cooking with both polluting fuels and LPG using their stoves more often, the average cooking time among households using both LPG and polluting fuels (3 h 19 min) was an hour less than that among exclusive polluting fuel users (4 h 10 min) (Fig. 1). Thus, households stacking LPG with polluting fuels may experience a marginal amount of time savings despite using their stoves more frequently.

### Socioeconomic status

We found lower SES to be associated with a longer mean daily cooking time and greater mean number of daily cooking events among households exclusively using polluting fuels (Table 3). This may be partly due to poorer households being more likely to have larger family sizes, which is known to increase stove use time due to larger meals. A greater cooking time among financially insecure households and those with lower levels of education may partially explain why lower SES is frequently associated with higher household air pollution levels^27^. A larger difference in mean daily cooking time between cooks exclusively using LPG and those only using polluting fuels among lower SES households suggests that time savings may be potentially greater when this subgroup transitions to clean cooking fuels. The social benefits that can accompany time savings, including increased income^11^, implies that expanding access to LPG for cooking may therefore help to lower economic disparities in LMICs. Additional interventional research is needed to confirm this hypothesis.

### Household air pollution monitoring

During the 24-hour period of household air pollution monitoring, the average daily stove use time among households using only polluting cooking fuels was over 1.5 h longer, on average, than the rest of the stove monitoring period in Eldoret and Mbalmayo (Table 2).

Moreover, households in Eldoret and Mbalmayo used their stove an average of one more time during the household air pollution monitoring period, compared with the rest of the SUM period (Table 2). This increase in stove use implies that participants may cook more frequently when they know their household air pollution exposures are being monitored. Thus, household air pollution levels in some measurement studies may be slightly higher than would occur on an average day, in the absence of air monitoring. This finding highlights the importance of clarity in messaging to participants when communicating that they should aim to cook as they normally would (i.e. ‘business as usual’) when being monitored for air pollution exposures.

Monitoring household air pollution levels over longer periods may also help mitigate the threat of measurement bias, as participants may be less likely to sustain their higher stove use frequency over longer time periods (as we found with the SUM data in our study). As air monitoring technology becomes increasingly more affordable and accessible, it will become more feasible to monitor household air pollution levels over longer time periods.

### Strengths and limitations

A key strength of our study is the use of objective temperature data to derive stove use measurements, rather than relying on participant self-report, which is prone to social desirability and response bias^28,29^. As we used the same SUMs under the same study protocol in all three settings, we were able to make multinational comparisons of stove use patterns. We collected multiple months of monitoring data to ensure that longer-term stove use patterns were captured; previous monitoring studies in sub-Saharan Africa have typically collected stove usage data over a period of a few days^30^.

As the FireFinder SUMS algorithm relies on abrupt changes in temperature within a 5-minute interval to identify the start and end time of a cooking event, the exact length of a stove use event may not be perfectly captured, introducing measurement bias. For example, cooking times among firewood stoves may be overestimate due to residual heat in fire pits that slows down the speed of temperature decline. However, given the substantial differences in reported average stove use times between households using LPG and those using only polluting fuels (on the order of 1 h or greater), we expect measurement error to not substantially impact the interpretation of our findings.

While it is possible that some households in our sample from Eldoret, Kenya (where the temperature is milder than the other two study communities) may have used their stove for heating, we took care to exclude households in Eldoret, Kenya that self-reported using their stoves for heating to increase the likelihood that all stove use reported in our analysis was due to cooking. More detailed survey data on heating times in communities with cooler climates is recommended for future studies to assess the proportion of stove use related to heating. Although we measured stove use patterns over several months, we did not monitor stove usage across all seasons. Therefore, our reported stove use patterns may not be representative of average annual cooking behaviors^24^. Nonetheless, this study presents one of the largest analyses of stove use patterns to-date, particularly in sub-Saharan Africa where a dearth of monitoring data is available.

Due in part to the use of stratified random sampling to select roughly equal numbers of households primarily cooking with LPG and exclusively cooking with polluting fuels, the proportion of exclusive LPG users in our dataset (30%) was much higher than what we found when conducting a population-based survey among a study sample of ~ 2,000 households in the study communities (4%). This may be also partly attributed to selection bias, as more affluent households that are exclusive LPG users may have been more willing to participate in the SUM and household air pollution monitoring phase of the study. Further, ‘fuel switching’ (households switching from partial to exclusive LPG use) may have occurred between the different phases of our study, which occurred several months apart. While the community-level stove usage statistics presented in our paper should be interpreted w/caution for these reasons, the cooking metrics presented within each fuel type are representative of usage. We also crosschecked the stoves monitored with SUMs in each household via surveys administered during the air pollution monitoring period to confirm that households were indeed exclusive LPG users. We are confident that all cookstoves were monitored with SUMs in study households.

We finally note that decreases in cooking time might not directly translate to time saved by the cook if they are not constantly engaged in the cooking process during a cooking event (e.g. occasionally stirring a pot versus constantly checking their food). Thus, our quantification of actual time saved by the cook may be an overestimate and should be interpreted cautiously. Nonetheless, the substantially lower cooking time (e.g. several hours) among LPG stoves relative to polluting fuels is likely to free up a meaningful amount of time for women, particularly when compounded over time since cooking is a daily activity.

## Conclusion

Our research has uncovered that households exclusively using LPG and using both LPG and polluting fuels spend substantially less time cooking than those exclusively using polluting fuels. Increased stove monitoring across a variety of communities in sub-Saharan Africa can improve our estimation of potential time savings associated with a large-scale transition from polluting cooking fuels to LPG for cooking.

We also found that access to LPG may help reduce SES disparities resulting from time poverty by reducing daily cooking time to a greater extent among lower income households. Finally, as greater-than-average levels of stove use occurred during HAP monitoring, clearly communicating the goals of monitoring to participants and conducting HAP monitoring over a longer period may help mitigate this issue.

## Data Availability

Data presented in this paper can be shared with others upon reasonable request made to the corresponding author.
